# Salt Intake of Children and Adolescents: Influence of Socio-Environmental Factors and School Education

**DOI:** 10.3390/nu16040555

**Published:** 2024-02-17

**Authors:** Ewa Malczyk, Małgorzata Muc-Wierzgoń, Edyta Fatyga, Sylwia Dzięgielewska-Gęsiak

**Affiliations:** 1 Department of Health Sciences and Physical Education, University of Applied Sciences in Nysa, 48-300 Nysa, Poland; ewa.malczyk@pans.nysa.pl; 2 Department of Public Health Silesian, Medical University of Silesia in Katowice, 40-055 Katowice, Poland; efatyga@sum.edu.pl (E.F.); sgesiak@sum.edu.pl (S.D.-G.)

**Keywords:** socio-cultural factors, school, salt intake, children, adolescents

## Abstract

(1) Background: The aim of this study was to investigate the salt consumption by children and adolescents from the Silesian Province (Poland), taking into account the region’s dietary traditions and the age of the students+. (2) Methods: 300 students aged 10–18 from different types of schools were enrolled in the study and divided into groups in terms of school, sex, and the state of their nutrition. A survey questionnaire about dietary habits, including the frequency and serving size with respect to 12 salty products, was used. On the basis of the frequency and the amount of consumed products, as well as the data on salt content, the amount of total daily intake of salt was estimated. (3) Results: The mean daily intake of salt by children and adolescents was 1.083 g (0.433 g of sodium); children aged 10–12 consumed the highest amount of salt (1.296 g/day) compared to pupils aged 13–15 (1.131 g of sodium) and adolescents aged 16–18 (0.863 g/day). (4) Conclusions: With age, as a result of various factors, the consumption of salt declines. The parents’ impact and the familial socio-environmental factors begin to wane, and other factors start to have influence, e.g., school education of a healthy lifestyle and health behavior of peers.

## 1. Introduction

Eating behaviors, also referred to as “food preferences”, are defined as “general predispositions to certain types of foods regardless of the situation in which they are eaten” and are conditioned by a range of various factors [1]. Food preferences comprise an integrated system, within which there are many interactions. Factors include stimuli, inhibitors, or specific conditions and affect food consumption [2,3,4]. Particularly significant are social factors (education level, type of occupation, job position, type of school, and family status), cultural factors (concerning the closest surroundings and being closely tied to the culture of the given country or region), and psychological factors [5,6].

The quick pace of social transformations, globalization, the imbalance in health (of societies and nations), migrations, and the formation of international cultures within social groups that have been hitherto homogeneous, all of which have been occurring in recent years, have led to changes in eating behavior patterns [2,4]. In Poland, eating habits that have existed in families for generations have started to undergo gradual modifications from the 1980s [7].

Silesia Province is a voivodeship in southern Poland, centered on the historic region known as Upper Silesia. In this region, the cuisine and the dialect have been two of the most important characteristics defining the identity of the Silesian ethnocultural group for generations. Their ethnic identity is expressed, for instance, in the everyday and occasional (holidays, anniversaries, and family events) preparation of dishes and their consumption, and these are long-existing customs. Traditional customs are cultural characteristics and, despite the support of previous (older) generations, are now undergoing modifications [8]. The analysis of the data available so far, particularly in the scope of a uniform area of studies concerning the Silesia Province, has encouraged us to discuss the possible impact of the local cultural heritage, developed over hundreds of years, and the power of tradition on the eating behaviors of the young generation [9,10]. In particular, this can be seen at the individual developmental stages of young people and the range of possible influence of other parts of social life changing over time, including upbringing, education, work, fashionable lifestyle, etc. The World Health Organization (WHO) suggests that health literacy should be incorporated in the core curriculum as children enter school, supported by a health-promoting school environment. A comprehensive school commitment towards students’ global wellbeing is expected to positively impact both children’s behaviors and their families [11,12].

Epidemiological studies show that the mean salt intake worldwide is estimated at 8–15 g/day. In Poland, it is estimated at 10–15 g/day; meanwhile, the daily intake recommended by WHO is 5 g/day, (i.e., 2 g/day of sodium) [13,14]. There is evidence that high salt intake by children and adolescents also contributes to elevated blood pressure [15,16] and may predispose an individual to the development of a number of diseases, e.g., cardiovascular diseases, mostly ischemic heart disease, brain stroke, hemorrhagic stroke and heart infarction, kidney disease (through increased urea concentration), osteoporosis and gastric cancer [17,18]. WHO recommends reducing sodium intake to control blood pressure in children aged 2–15 years. These recommendations recognize that salt reduction and salt iodization are compatible [19]. According to the Centers for Disease Control and Prevention (CDC), about 9 in 10 children consume more sodium than recommended [20]. The main source of excess sodium is processed foods: the top 10 sources of sodium for Americans ages 6-18 are pizza, mixed Mexican dishes, sandwiches, bread and rolls, cold cuts and cured meats, soups, savory snacks, cheese, plain milk and poultry. The recommendations of the European Food Safety Authority Panel on Nutrition, Novel Foods and Food Allergens for a daily sodium intake that is safe and adequate for children are extrapolated from values for adults, taking into account their energy requirements and growth factors, and are as follows: 1.1 g/day for children aged 1–3 years, 1.3 g/day for children aged 4–6 years, 1.7 g/day for children aged 7–10 years and 2.0 g/day for children aged 11–17 years, respectively [21].

When Hu et al. analyzed the effects of high salt diet on gut microbiome, they demonstrated a considerable decrease in Bacteroidetes and Proteobacteria by 50.53% and 2.96%, respectively, and an increase in Firmicutes by 42.77% versus a healthy composition of gut microbiota [22]. In view of concerning reports regarding excessive salt consumption in Poland [13,23,24], the present study aims to assess the salt (sodium chloride) intake of school-age children and adolescents from the Silesian Province (Poland), taking into account the region’s dietary traditions [25] and the age of the students.

## 2. Materials and Methods

### 2.1. Ethics

The current study protocol was registered with the Bioethical Committee of the Medical University of Silesia in Katowice. The Committee wrote that “the project does not meet the criteria of a medical experiment in the context of law and does not require assessment by the bioethical committee”. Still, according to the Declaration of Helsinki 2013, participation in the study was voluntary and informed. Moreover, participants were informed in detail about the study—the study was preceded by informative meetings with the school heads and letters were sent to the respondents’ parents or guardians outlining the purpose, scope and methodology of the project. Written consent was obtained from the directors and parents or guardians of the children/adolescents and from the student if they were aged 16 or over. Thus, patient data were encoded in accordance with the pseudo-anonymization procedure, which means that personal data were processed in such a way that it could not be assigned to a specific data subject without the use of an additional “key”.

### 2.2. Participants

Three hundred pupils aged 10–18 years from different types of schools in a small Silesian city (elementary, lower secondary, and higher secondary schools) were included in the study. The inclusion criteria were obtaining written consent to participate in the study.

The study consisted of four phases.

Phase one was the development and validation of the authors’ questionnaire and this began with an initial draft list of the questionnaire items generated by the authors based on the literature. A pilot study had been previously conducted twice on a group of 45 children (15 from each age subgroup). The interval between the first and second survey was 30 days, according to the arbitrary criteria used to assess validity of the research tool. Based on literature data on children’s preferred snacks and interviews with the children surveyed, 20 products were preselected and evaluated in the questionnaire validation process. A question about snacking preferences (“What snacks do you eat most often?”) was answered based on a 4-point agreement option (1—never; 2—rarely; 3—often; 4—very often). Higher scores indicated children’s preferences. High or low levels were classified according to the median grouping of the dimension score.

The final questionnaire contained the following: (a) questions about sex, age, body weight, body height, type of school, and (b) questions regarding eating behavior, taking into account frequency and sizes of helpings of the 12 salt products.

Children aged 10–15 completed the questionnaire in the presence of their parents.

Based on national surveys [11,12] and a pilot study, we analyzed the following 12 products: salted crisps, unsalted crisps, salted biscuits, unsalted biscuits, salted sticks/pretzels, unsalted sticks/pretzels, corn curls, salted peanuts, unsalted peanuts, batter-coated peanuts, popcorn, and roasted salted sunflower seeds. The respondents could choose between the following frequency criteria: several times a day (<3 times, 3 times, 2 times, and 1 time), several times a week (1 time, 2 times, 3 times, 4 times, 5 times, and 6 times), several times a month (1 time, 2 times, and 3 times), rarely (up to 5 times a year and over 5 times a year), and no consumption. Portion sizes of the accepted products were determined by the children during the initial interview and validation of the questionnaire. In order to determine the portion size of the products consumed, the survey included original photos of portions of the products modeled according to the Album of Photographs of Food Products and Dishes [26]. The album of 201 color photos, prepared and published by the Food and Nutrition Institute in Poland, is a popular tool used in this type of research. Based on the album, pictures were taken of all products according to the portion size obtained from the validation of the questionnaire. To help to determine the portion sizes consumed by respondents, several photos of different portions sizes of products were prepared. The following helping sizes of products were assumed: for crisps, 20, 40, and 70 g; for biscuits, 10, 20, and 30 g; for sticks, pretzels, and corn curls, 10, 20, and 50 g; for peanuts and batter-coated peanuts, 20, 30, and 50 g; for popcorn, 10, 20, and 30 g; and for roasted salted sunflower seeds, 10, 20, and 40 g. This allowed children to look at the pictures and choose the right product and the portion (amount) they consumed. The amount of salt in the analyzed products was taken according to the posted information on the package (e.g., salted chips contained 1.78 g of salt per 100 g of product, popcorn—2.64 g/100 g). The salt content in the above products ranged from 0.93 g per 100 g in salted peanuts to 4.27 g per 100 g in salted sticks. The mean was 2.05 g per 100 g of product.

During phase two, the questionnaire devised by the authors was distributed to the pupils with informed consent and they were given a deadline to return them by. Phase three was determining which pupils had returned their properly completed questionnaires, which limited participation in the study.

During phase four, on the basis of the frequency and the helping size of the consumed products and on the data on salt content found on product packages, the amount of the total daily intake of salt with a diet was estimated.

The estimated daily intake (EDI) of salt was calculated according to the following formula, depending on the helping size:

EDI = F/100 × M × R [g/day]

where EDI is the estimated daily intake of salt; F is the data on the helping size of product [g]; M is the salt content in products [g/100 g of product]; R is the data on the frequency of intake converted into days (multiple daily consumption—R/1, several times a week—R/7, several times a month—R/30, rarely—R/365; e.g., 3 times a day: 3/1; weekly—e.g., 5 times a week: 5/7; Monthly—e.g., 2 times a month—2/30).

For example, F = 20 g salt chips, R = frequency of consumption, e.g., 5 times a week, or 5/7, M = 1.79 g/100 g of product—EDI = 20 g × 1.79 g/100 g × 5/7 = 0.255 g salt/day.

For the purposes of the analysis, the respondents were divided into groups in terms of the type of school, sex, and the state of their nutrition. The state of nutrition was assessed with percentile charts. The BMI centile charts developed in 2010 by the Children’s Health Centre in Warsaw were used to interpret the results and these provided the following BMI definitions: underweight: <5th percentile; normal weight: ≥5th percentile to <85th percentile; overweight: ≥85th percentile to <95th percentile; and obesity: ≥95th percentile [27].

On the basis of the estimated intake of salt from the salty snacks, the amount of sodium intake was calculated, given that 5 g of salt (NaCl) provides 2 g of sodium (Na). In addition, the percentage of the daily sodium requirement coverage was calculated given that the mean norm for the studied group was 1400 mg and that, for individual age groups, it was 1300 mg (10–12 year olds) and 1500 mg (13–18 year olds) [13,14].

### 2.3. Statistical Analysis

The statistical analysis was conducted with Statistica v.13.0. A hypothesis about the normal distribution of analyzed variables was verified with the Shapiro–Wilk test. To demonstrate the difference in the amount of salt consumed with diet, taking into account the type of school (age), the sex, and the state of nutrition (according to BMI), the Mann–Whitney U test, the Kruskal–Wallis test (distribution other than normal distribution), a one-way analysis of variance (one-way ANOVA), or the Tukey procedure (normal distribution) were conducted. A *p*-value of *p* < 0.05 was statistically significant.

## 3. Results

The total sample group consisted of 300 students, including 165 girls and 135 boys. The students were divided into three age groups: 10–12, 13–15 and 16–18 years. The largest group was from the upper grade of elementary school (38%), followed by high school (35%) and elementary (27%) school. Normal body weight was found in 251 (84%) pupils, underweight in 31 (10%), overweight in 15 (5%), and obesity in only 3 (1%)(Table 1).

### 3.1. Salt/Sodium Intake Estimation

The mean daily intake of salt from salty snacks by children and adolescents was 1.083 g (0.433 g of sodium). The consumption of salty snacks covered nearly 31% of the daily requirement for that mineral (given that the norm for the non-homogeneous study group was 1.4 g/day). The statistical analysis showed the salt intake differed depending on the sex of the respondents (*p* < 0.05). The boys consumed 1.423 of salt (i.e., 0.569 g of sodium) daily, and girls consumed 0.809 g salt (i.e., 0.324 g of sodium). The maximum salt intake by the boys was 10.247 g/day (i.e., 4.1 g of sodium per day) and that by the girls was 8.513 g/day (i.e., 3.4 g of sodium per day)—Table 2. The salty snacks consumed by the boys covered over 40% of the daily requirement for sodium.

The age of the respondents did not determine the amount of the salt consumed in diet. However, a certain tendency was observed. Pupils aged 10–12 consumed the highest amounts of salty snacks, which was reflected in the intake of the highest amount of salt (1.296 g/day, i.e., 0.518 g of sodium per day) compared to pupils aged 13–15 (1.131 g/day, i.e., 0.452 g of sodium per day) and pupils aged 16–18 (0.863 g/day, i.e., 0.345 g of sodium per day).

Among the youngest respondents, as well as among people with underweight, 65% reported the highest frequency of consumption of salty snacks (at least once a week). The products of choice with such frequency were the salted sticks or salted pretzels. Students with obesity frequently consumed several types of salty snacks, such as salted sunflower seeds, salted sticks or salted pretzels, corn curls and popcorn (several times a week to several times a month).

Similarly, the shares of sodium from salty snacks for elementary, lower secondary, and higher secondary school students were 39.9, 30.2, and 23.0% of the daily requirement for that mineral, respectively.

The state of nutrition of all pupils, determined according to BMI, was the factor differentiating the mean amount of salt intake. The highest salt intake was observed in the obese pupils—2.896 g/day (i.e., 1.158 g of sodium/day)—and the lowest in overweight pupils—0.790 g/day (0.316 g of sodium/day). The pupils who were normal and underweight consumed similar amounts of salty snacks and in turn consumed similar amounts of sodium chloride intake (1.059 and 1.232 g, respectively) and sodium intake (0.424 and 0.493 g, respectively).

#### 3.1.1. Analysis of Elementary School Pupils

Sex differentiated the amount of salt intake of elementary school pupils. The girls’ intake was 0.985 g of salt/day (i.e., 0.394 g of sodium/day) and the boys’ intake was 1.614 g (i.e., 0.646 g of sodium/day) (Table 3).

Through consuming salty snacks, the boys covered almost half of their daily requirement of sodium (49.7%). Age did not differentiate the intake of salt (sodium). The mean daily intake of sodium in salty snacks among 10–12-year-olds was similar and ranged from 1.097 to 1.388 g, and teenagers’ daily intake of sodium ranged from 0.439 to 0.555 g. However, it should be noted that, with age, the share of salt (sodium) from consumed salty snacks increased (33.8, 39.3, and 42.7%).

Among elementary school pupils, the state of nutrition based on BMI did not differentiate the amount of salt intake. The respondents’ salt intake ranged from 1.181 to 1.311 g per day (i.e., 0.472–0.524 g of sodium).

#### 3.1.2. Analysis of Upper Grade of Elementary School Pupils

Among pupils, sex was the factor differentiating the intake of salt (Table 4). The boys’ intake of sodium chloride was higher than the girls’ intake (1.540 and 0.762 g/day, respectively) and the difference was statistically significant. The share of salt from salty snacks in covering the daily requirement for sodium was twice as high in the boys (41.1 vs. 20.3%).

Age did not differentiate the amount of consumed salt. The highest amount of salty snacks was consumed by 15-year-old pupils, which was reflected in the intake of the highest amount of salt (1.471 g/day; 0.855 g of sodium per day). The lowest daily intake of sodium chloride in that group was exhibited by 13-year-olds. The share of salt from salty snacks in covering the daily requirement for that ingredient was distributed in the same way (39.2 vs. 27.3 vs. 21.9%). The mean daily intake of salt was not statistically different given the various states of nutrition based on BMI. The highest salt intake was exhibited in underweight respondents (1.210 g/day), and the lowest amount of salty snacks was eaten by overweight and obese respondents. Salt intake among those pupils ranged from 0.175 to 0.242 g/day.

#### 3.1.3. Analysis of High School Pupils

Sex did not determine the intake of salt from salty snacks by higher secondary school pupils. The intake of that ingredient by the girls, i.e., 0.741 g/day, was slightly lower compared to the boys (1.065 g/day)—see Table 5. Nevertheless, the share of salt resulting from the consumption of salty snacks in the girls’ diet was nearly 9% lower compared to the boys’ diet. The salt intake by pupils was differentiated by age (*p* < 0.05). The highest amounts of sodium chloride were consumed by 16-year-olds. The daily intake of salt in that age group was nearly three times higher than in the 18-year-old group (1.359 and 0.488 g, respectively). Similarly, the share of salt in the diet of 16-year-olds was three times higher than the share of salt in the diet of 18-year-olds and two times higher compared to the diet of 17-year-olds.

The higher secondary school pupils’ salt intake was determined by the state of nutrition based on BMI. The mean daily intake of sodium chloride by obese pupils was as much as 4.257 g, thus exceeding the daily requirement for that compound by 13.5%. The salt intake of these respondents was nearly six times higher compared to the intake of the young people with a normal body weight.

## 4. Discussion

Many studies have indicated that food preferences are a complex of psychological, social, and cultural factors [2,3]. The impact of these factors is different at different life stages. In childhood, the family (“family environment”) and close relations formed are the most significant and at school age and during puberty, the peer group is key for the development of young people. In the case of adults, mass media, reference groups, fashion, or prestige are the relevant factors [28]. School is the second environment, coming next after family house, that influences a proper lifestyle, including the nutrition of children and adolescents [29].

### 4.1. Salty Snack Consumption Frequency

Our study indicated that 65% surveyed children and young people ate salty snacks at least once a week, most often when meeting with friends, watching TV, studying, or going to the cinema. Inhabitants of other European countries ate salty snacks at a similar frequency [30]. The highest level of salty snack consumption was exhibited by Spaniards (78%) and then by the population of Great Britain (76%). In the cited studies, the percentage of Poles (regardless of age and sex) eating salty snacks at least once a week was 66%. Similar results were obtained in the studies of adolescents living in the Silesian Region, where 70% of adolescents snacked at least once a week, between meals [31], while Jensen et al. [32] concluded that 95.2% of Chilean children and 89.9% of adolescents consumed at least one snack per day, with a mean per capita of 2.30 ± 0.03 snacks consumed per day.

These analyses highlight that pupil groups regularly eat salty snacks between meals. Several studies have showed that salt preference might be associated with the frequency or the amount of salty food consumption [33,34].

### 4.2. Salt/Sodium Intake

In Poland, no social group consumes just 5 g of salt daily. The most salt, as much as 16 g per day, is consumed by pensioners. The lowest amount of salt is consumed by the self-employed (8.8 g of salt per day) [13,27,35].

The present study shows that pupils attending the schools of Silesian city consume 1.083 g of salt/day, i.e., 0.433 g of sodium per day, through salty snacks alone. According to Ponzo et al. [36], 11–13-year-olds obtain much more sodium from snacks. The mean consumption of sodium from snacks was 1.4 g/day. On the basis of analysis of our own results, it was determined that boys consumed significantly more sodium compared to girls (0.569 g of sodium/day vs. 0.324 g of sodium/day, respectively). A similar correlation was indicated in a Polish salt consumption report. The salt consumption of boys over 13 and men from 19 to 25 years, who preferred salty products, reached 15 g per day. Girls aged 13–15 years consumed 10 g of salt on average [13]. The study by Strohm et al. [37] estimated the sodium intake in children and adolescents ranging from 400 mg sodium/day to 1500 mg/day (for teenagers aged 15–19). According to the American Heart Association [18], boys aged 12–19 eat the most sodium—an average of 4220 mg/day; girls at the same age eat about 2950 mg/day.

Studies of Spanish children between 7 and 11 years old have shown that boys consume more dietary sodium and sodium from ultra-processed food (UPF) than girls. The main sources of dietary sodium were meat and meat products (25.1%), ready-to-eat and pre-cooked dishes (7.4%), and sugars and sweets (6.3%) [38].

Whether or not a child’s eating habits will be healthy depends to a high extent on the sociocultural environment where they grow up. Their first educators are their parents, then their siblings and family, and subsequently their teachers. The fashion or the promoted lifestyle also has a considerable impact in this area. The prices of healthy vs. unhealthy foods are also significant.

We observed that children aged 10–16 consume the highest amounts of sodium: from 0.328 to 0.588 g per day. On the other hand, 17- and 18-year-olds obtain salty snacks much less often, which implies a lower salt intake compared to other pupils (0.195–0.282 g/day).

### 4.3. Risks of a High Salt Intake in Children

There is evidence that a high-salt diet during childhood and adolescence may increase the development of many diseases in adults. For example, Leyvraz et al. [39], Wirix et al. [40], and Emamian et al. [41] found a positive association between sodium intake and blood pressure in children, which increases the risk of heart disease and strokes threefold in the future [42]. A meta-analysis of six studies of children with elevated blood pressure (BP) without an identifiable cause showed a difference of 6.3 mm Hg (95% CI 2.9–9.6) and 3.5 mm Hg (95% CI 1.2–5.7) in terms of systolic and diastolic BP, respectively, for each additional gram of sodium per day [43]. Studies by Ma et al. [44] and Yoo et al. [45] showed that baseline salt intake was positively associated with an increase in body fat percentage, regardless of energy intake. It is likely that a high-sodium diet causes obesity by stimulating thirst and inducing higher adiposity. Other potential health effects of high-sodium diets include osteoporosis ( increase in salt in urinary calcium excretion), autoimmune diseases (dietary salt intake has been shown to affect immune cell function and causes a pro-inflammatory response) and stomach cancer [46].

### 4.4. Parental Habits Related to Salt Intake

It might be ventured that a higher salt intake from salty snacks among younger children results from their parents’ lack of eating awareness. Parents have the greatest influence on their children’s dietary choices: they decide what products and dishes their children will eat [3], but parents’ influence decreases with the transition from childhood to adolescence [47]. According to the Identification and prevention of Dietary- and lifestyle-induced health Effects in Children and infants (IDEFICS) study, which involved 1435 families from eight European countries, the food environment at home plays a greater role in influencing children’s consumption of healthy foods than unhealthy products, especially for younger children [48]. Regarding sodium intake, among Spanish school-age children, parent and child iodized salt and table salt intake and the presence of a salt shaker on the table were associated with children’s sodium intake [49]. Szajewska et al. [50] documented that salt consumption is excessive in 90% of children under 3 years of age. It was found that this is not due to a greater desire to eat salty products (e.g., crisps), but is the fault of adults who believe that dishes served to their children must be properly salted. This belief of the parents is then “passed on” to the young generation. Four groups were conducted in Belgium among parents/caregivers, showing that the influence of parental practices differs by age—the younger the child the stronger the role of parental practices.

As noted above, with age, the consumption of salty snacks declines as a result of various factors (higher eating awareness and school education), The parents’ impact starts to wane, and factors outside the family start to play a role, e.g., above all, the school that promotes healthy habits and healthy lifestyle trends, peers, and the media.

In many countries, 80% of consumed salt comes from processed products such as bread, breakfast cereals, ready-made dishes and snacks, and dishes served in restaurants, canteens, cafes, or other food premises. It was determined that children cover 15% of their daily requirement for sodium at breakfast, 30% at lunch, and 39% at dinner, and 16% of their sodium intake comes from salty snacks [51,52].

In our studies, the sodium intake from salty snacks by the pupils covered nearly 31% of the daily requirement of sodium. This coverage differed at different stages of ontogenetic development. Ponzo et al. [36] concluded that the consumption of sodium from salty snacks among teenagers comprised nearly half of the mean daily intake of sodium. Based on the present study, the amount of consumed salt, and in turn sodium, is very high, as daily sodium intake includes other foods that are eaten and that contain that element, such as bread, highly processed breakfast cereals, meat products, yellow cheeses, cheese spread, margarine, and even sweets.

#### Strengths and Limitations

The questions were developed on the basis of large, international studies on the nutritional behavior of school-age children [53]. All questionnaires were completed under the supervision of a trained dietician, who was able to clarify the respondents’ doubts on an ongoing basis.

Respondents for our study were recruited from schools in one Silesian city, which may constitute a certain limitation. Another limitation of the study is the sample size—300 students—and the lack of a parallel survey of parents and even grandparents due to the traditions of Silesian families. Simultaneously, there were no additional data on sodium homeostasis, including total caloric intake, past medical history, or medications taken, e.g., diuretics. Variations in parental income, education, and the non-division of families between so-called native Silesians and immigrants may also significantly affect children’s food choices and preferences, introducing a layer of complexity not addressed in the current investigation.

## 5. Conclusions

In the present study, children and adolescents of the Silesian voivodship aged 10–18 enjoy and consume salty snacks on a daily basis, despite the fact that Silesian cuisine is one of the most distinctive and tasty regional cuisines.

In Poland, the consumption of salty snacks covered almost 31% of the daily requirement for this mineral, and the mean daily intake of salt in salty snacks by children and adolescents aged 10–18 was 1.083 g (0.433 g of sodium).

It was found that salt intake varies according to the age (type of school) and sex of respondents, and these relations were not symmetrical. The mean daily salt intake was not statistically different taking into account various states of nutrition based on BMI. Pupils aged 10–12 consumed the highest amounts of salty snacks, which was reflected in their intake of the highest amount of salt, while the least salty snacks were consumed by youths aged 16–18. It is likely that parents accept their children consumption of salty snacks, despite the variety of flavors of Silesian dishes. At this age, young people create their own eating behaviors, dependent on their school education, peer group, a lifestyle that promotes healthy habits and healthy lifestyle trends, peers, and the media.

Practical implications arising from the above data mainly concern the following:

1. The need for ongoing nutrition education of children, parents, grandparents about healthy eating and salt restriction in main meals and snacks. Teaching children and adults to make informed food choices; e.g., apple or salty pretzels? Nutrition education in the form of lectures and workshops (e.g., family preparation of healthy snacks).
2. While implementing pt. 1, we should at the same time develop a sense of responsibility for the health of loved ones. Not only, for example, parents to children, but also vice versa.
3. The need to pay attention to the composition of purchased products—reading information on packaging (conducting thematic information campaigns in schools and the media).

## Figures and Tables

**Table 1:** Characteristics of the studied group.

Total		N	%
		300	100
Sex	Girls	165	55
	Boys	135	45
Age/school	10–12/elementary	81	27
	13–15/upper grade of elementary	114	38
	16–18/high school	105	35
BMI	Underweight	31	10
	Normal body weight	251	84
	Overweight	15	5
	Obesity	3	1

BMI calculated according centile charts by Kułaga et al. [27].

**Table 2:** The mean amount of the intake of salt (sodium) (g/day) by the pupils, taking into account the sex, age, and the state of nutrition; % of daily requirement for sodium according to age and sex norms.

		NaCl	Na	% of Recommended Daily Intake of Na Covered
	Total, M (SE)	1.083 (0.09)	0.433 (0.04)	30.9
Sex *	Girls, M (SE)	0.809 (0.09)	0.324 (0.04)	23.1
	Boys, M (SE)	1.423 (0.17)	0.569 (0.07)	40.7
Age [years]	10–12, M (SE)	1.296 (0.17)	0.518 (0.07)	39.9
	13–15, M (SE)	1.131 (0.18)	0.452 (0.07)	30.2
	16–18, M (SE)	0.863 (0.12)	0.345 (0.05)	23.0
State of nutrition *	Underweight, M (SE)	1.232 (0.24)	0.493 (0.09)	35.2
	Normal body weight, M (SE)	1.059 (0.10)	0.424 (0.04)	30.3
	Overweight, M (SE)	0.790 (0.24)	0.316 (0.10)	22.6
	Obesity, M (SE)	2.896 (2.86)	1.158 (1.14)	82.7

*—statistically significant differences at *p* < 0.05; M: mean; SE: standard error.

**Table 3:** The mean amount of salt intake (g/day) by elementary school pupils, taking into account sex, age, and the state of nutrition (norm for 10–12-year-olds: 1.300 g).

		N	NaCl	Na	% of Recommended Daily Intake of Na Covered
	Total, M (SE)	81	1.296 (0.17)	0.518 (0.07)	39.9
Sex *	Girls, M (SE)	41	0.985 (0.16)	0.394 (0.06)	30.3
	Boys, M (SE)	40	1.614 (0.30)	0.646 (0.12)	49.7
Age [years]	10, M (SE)	17	1.097 (0.27)	0.439 (0.11)	33.8
	11, M (SE)	23	1.278 (0.30)	0.511 (0.12)	39.3
	12, M (SE)	41	1.388 (0.28)	0.555 (0.11)	42.7
State of nutrition	Underweight, M (SE)	7	1.181 (0.30)	0.472 (0.12)	36.3
	Normal body weight, M (SE)	71	1.311 (0.19)	0.524 (0.07)	40.3
	Overweight, N, (SE)	3	1.212 (0.70)	0.485 (0.27)	37.3

*—statistically significant differences at *p* < 0.05; M: mean; SE: standard error.

**Table 4:** The mean amount of salt (sodium) intake (g/day) in upper grade elementary school pupils, taking into account sex, age, and the state of nutrition (norm for 13–15-year-olds: 1.500 g).

		N	NaCl	Na	% of Recommended Daily Intake of Na Covered
	Total, M (SE)	114	1.131 (0.18)	0.452 (0.07)	30.2
Sex *	Girls, M (SE)	60	0.762 (0.16)	0.305 (0.06)	20.3
	Boys, M (SE)	54	1.540 (0.33)	0.616 (0.13)	41.1
Age [years]	13, M (SE)	17	0.820 (0.48)	0.328 (0.19)	21.9
	14, M (SE)	62	1.024 (0.22)	0.410 (0.08)	27.3
	15, M (SE)	35	1.471 (0.40)	0.588 (0.16)	39.2
State of nutrition	Underweight, M (SE)	9	1.210 (0.54)	0.484 (0.21)	32.3
	Normal body weight, M (SE)	99	1.178 (0.21)	0.471 (0.08)	31.4
	Overweight, M (SE)	5	0.242 (0.08)	0.097 (0.03)	6.5
	Obesity, M (SE)	1	0.175	0.070	4.7

*—statistically significant differences at *p* < 0.05; M: mean; SE: standard error.

**Table 5:** The mean amount of salt (sodium) intake (g/day) by higher secondary school pupils, taking into account sex, age, and the state of nutrition (norm for 16–18-year-olds: 1.500 g).

		N	NaCl	Na	% of Recommended Daily Intake of Na Covered
	Total, M (SE)	105	0.863 (0.12)	0.345 (0.05)	23.0
Sex *	Girls, M (SE)	64	0.741 (0.16)	0.296 (0.06)	19.8
	Boys, M (SE)	41	1.065 (0.17)	0.426 (0.07)	28.4
Age [years] *	16, M (SE)	33	1.359 (0.32)	0.544 (0.13)	36.2
	17, M (SE)	46	0.704 (0.12)	0.282 (0.05)	18.8
	18, M (SE)	26	0.488 (0.12)	0.195 (0.05)	13.0
State of nutrition *	Underweight, M (SE)	15	1.268 (0.37)	0.507 (0.15)	33.8
	Normal body weight, M (SE)	81	0.690 (0.10)	0.276 (0.04)	18.4
	Overweight, M (SE)	7	1.036 (0.41)	0.414 (0.16)	27.6
	Obesity, M (SE)	2	4.257 (4.06)	1.703 (1.62)	113.5

*—statistically significant differences at *p* < 0.05; M: mean; SE: standard error.

## Data Availability

The data presented in this study are not publicly available. Data are available on request from the corresponding author.
